# Translation Microscopy (TRAM) for super-resolution imaging

**DOI:** 10.1038/srep19993

**Published:** 2016-01-29

**Authors:** Zhen Qiu, Rhodri S Wilson, Yuewei Liu, Alison R Dun, Rebecca S Saleeb, Dongsheng Liu, Colin Rickman, Margaret Frame, Rory R Duncan, Weiping Lu

**Affiliations:** 1 Institute of Biological Chemistry, Biophysics and Bioengineering, Heriot-Watt University, Edinburgh, EH14 4AS; 2 Edinburgh Super-Resolution Imaging Consortium, Heriot-Watt University, Edinburgh, EH14 4AS; 3 Department of Chemistry, Tsinghua University, Beijing, China; 4 University of Edinburgh, Western General Hospital, Crewe Road South, Edinburgh, EH4 2XR

## Abstract

Super-resolution microscopy is transforming our understanding of biology but accessibility is limited by its technical complexity, high costs and the requirement for bespoke sample preparation. We present a novel, simple and multi-color super-resolution microscopy technique, called translation microscopy (TRAM), in which a super-resolution image is restored from multiple diffraction-limited resolution observations using a conventional microscope whilst translating the sample in the image plane. TRAM can be implemented using any microscope, delivering up to 7-fold resolution improvement. We compare TRAM with other super-resolution imaging modalities, including gated stimulated emission deletion (gSTED) microscopy and atomic force microscopy (AFM). We further developed novel ‘ground-truth’ DNA origami nano-structures to characterize TRAM, as well as applying it to a multi-color dye-stained cellular sample to demonstrate its fidelity, ease of use and utility for cell biology.

In optical microscopy, image resolution is limited by the standard diffraction limit^1^, of about 200 nm for visible light. Resolutions that exceed this limit are referred to commonly as super-resolution in light microscopy. Three main approaches have been developed to achieve this. First, hardware-based technologies aim to shape the point spread function (PSF), such as in STED^2^ or nonlinear structured illumination microscopy (SIM)^3^, that employs optical patterning of the excitation and a nonlinear response of the sample. Second, biological and software technologies, grouped together as single molecule localization microscopy (SMLM)^4^, try to image single PSFs separated in time, calculating the positions of the single molecules that give rise to the signals with a precision certainty substantially better than the diffraction limit. A super-resolution map is then reconstructed by projecting together all the individual measurements acquired at different time points^5^,^6^,^7^. The third approach is computational, in which image processing techniques are employed to restore a super-resolution image from a set of low-resolution observations^8^. In this approach, a low-resolution observation is considered as the outcome of a degrading process of a high-resolution image due to blurring and noise effects. The super-resolution restoration method is therefore a post-acquisition inverse process. The accessibility of all these super-resolution microscopy approaches is limited currently, as all three general modalities require a high degree of technical expertise and a need for sample preparation to match the modality employed. Furthermore, there is an acute requirement in biology for a simple route towards multi-color super-resolution imaging; this is currently technically difficult in almost all current super-resolution modalities.

Recently, there has been a substantial effort to develop super-resolution restoration techniques for medical imaging, such as X-ray mammography, functional magnetic resonance imaging (fMRI) and positron emission tomography (PET)^9^. Medical imaging usually uses highly controlled illumination doses to avoid damage to the subject^9^, leading to low signal-to-noise ratio (SNR) images. Noise removal therefore becomes critically important for the performance of super-resolution restoration^9^,^10^. However, there is a trade-off between noise removal and feature preservation; over-smoothing impedes the image resolution that can be restored^11^. To date, the prior models for regularizing noise reduction have usually been constructed based on the edge-preservation concept in medical and other applications^10^; features are restored as long as the edges are preserved in the inverse process. Biological imaging is often more challenging than medical imaging in terms of complexity and feature size. Medical images contain data describing tissues typically 2-3 times smaller than the resolution-limit of the system^9^, whereas fluorescence images of intracellular structures contain abundant, heterogeneous features, and complex sub-cellular structures, potentially many orders-of-magnitude smaller than the diffraction-limit^12^. In general, edges embedded in such small and complex features are more prone to noise contamination, leading to poor performance of edge-based methods in fluorescence microscopy images^13^. Prior models based on edge-preservation therefore cannot fully capture these structures in fluorescence biological microscopy due to their complexity and low SNR environments. As such, super-resolution (SR) restoration methods using these models do not perform well in fluorescence biological imaging.

Here, we present a novel super-resolution method we call translation microscopy (TRAM). In TRAM, a super-resolution image is restored, using signal processing techniques, from a set of diffraction-limited low-resolution images acquired whilst moving (translating) the sample in the XY plane, as depicted in Fig. 1a. An advantage of TRAM is that these movements can be in multiple directions, and with non-exact step-sizes, allowing small regions of interest to be scanned without leaving the field of view. In contrast to the existing super-resolution restoration methods for medical imaging, here we explore a sophisticated prior model that is capable of preserving complex and fine biological structures in the inverse process. Distinct from other super-resolution imaging modalities, TRAM can be performed using any microscope equipped with a movable stage with no modification to hardware or sample preparation methodology. We show that TRAM can achieve up to a 7-fold increase in lateral resolution in a cellular environment. Our results were compared with state-of-the-art gSTED microscopy, in this case measuring resolution using sub-diffraction nanobeads^14^ and correlation with atomic force microscopy (AFM)^15^ data using novel DNA origami-quantum dot nano-structures^16^ to provide a reliable ‘ground-truth’. In addition, we used an off-the-shelf, multi-color organic dye-stained biological sample selected because it had not been subjected to any specialized preparation protocols, demonstrating a large improvement in resolution and contrast compared to confocal laser-scanning microscope (CLSM).

## Results

**Figure 1:**
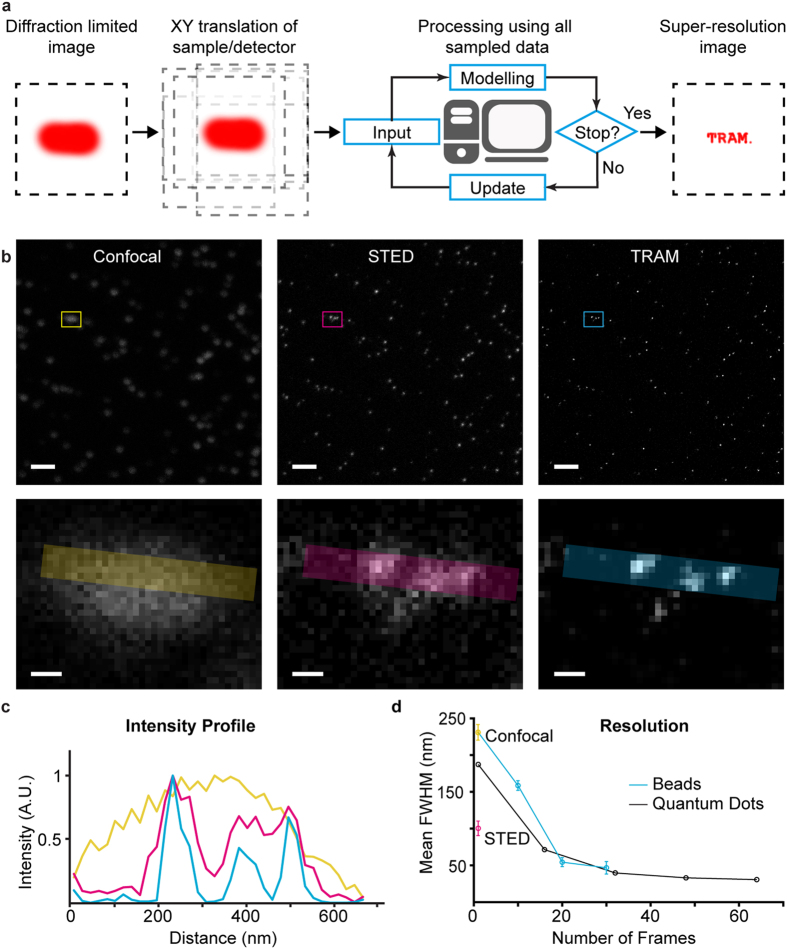
Translation microscopy delivers super-resolution image data in a simple, inexpensive way. (**a**) Schematic illustration of translation microscopy (TRAM). It involves acquiring multiple images of the same sample, as the object is moved on the microscope stage. All the acquired data are then used to rationally denoise and restore a super-resolution image through an iterative energy minimization process. (**b**) Top row: Confocal, gSTED and TRAM fluorescent bead images (from left to right). There are more than 100 bright spots observed in the confocal image. Scale bar 1 μm. Bottom row: A zoom area of (*left to right*) CLSM, gSTED and TRAM 40 nm-diameter fluorescent bead images from the same field of view. Scale bar 100 nm. (**c**) Line intensity profiles of the same region from the three images, showing an improvement in contrast and resolution across three closely adjacent objects. (**d**) Spatial resolution *versus* the number of translation images used for each TRAM restoration for the beads data presented here and quantum dot images in Fig. 2a.

First, we tested TRAM on a range of image datasets, both simulated and real (Supplementary Figs S1-S2). Here we focus on four-exemplar fluorescence datasets: fluorescent beads, quantum dots, quantum dots on a DNA origami scaffold and stained pulmonary endothelial cell. First, 40 nm-diameter fluorescent bead (i.e., sub-resolution) image data were acquired using a CLSM (pixel size of 20 nm), whilst translating the sample 30 times in the imaging plane in the order of 100 nm-steps in an XY-grid pattern. We applied our TRAM restoration method to these images. We acquired images from the same field of view using gSTED to allow direct assessment of results from CLSM, gSTED and TRAM (Fig. 1b: top row). As seen from the full view of the these images, there are more than 100 bright spots observed in the confocal image. TRAM shows the capability of separating adjacent beads, as gSTED does, the two are shown to have a one to one correspondence between each spot. We analyzed the data in detail from a small region-of-interest (ROI) for each modality (Fig. 1b: bottom row), finding that as expected, gSTED could resolve beads underneath the diffraction limit (*middle* panel), that were not detectable as separate objects in the CLSM image (*left* panel). TRAM performed favorably (*right* panel), delivering lateral resolution well below the diffraction-limit. Quantifying this data using line-plots confirmed that TRAM could resolve closely adjacent structures that were not resolvable using CLSM (Fig. 1c). Figure 1d shows the mean values of the full width at half maximum (FWHM) of the Gaussian-fitted (n = 20) beads intensity profiles in the full field of view, which is commonly used as an estimate of the lateral resolution: confocal ~231 nm and TRAM restorations using different numbers of translation images: 10 images ~158 nm, 20 images ~55 nm and 30 images ~47 nm. The scaling law (Fig. 1d) on increasing TRAM resolution with the number of translation images is consistent with the quantum dot experiment (see below) and also synthetic data in Supplementary Figs S1 and S2. For gSTED, while the non-optimized measurement is ~100 nm, further optimization of the STED conditions resulted in superior results compared to TRAM in this test (the mean value of FWHM is 44 nm, Supplementary Fig. S3). Nevertheless, our finding that TRAM can deliver sub-diffraction resolution data is indicative of the utility, relative simplicity, and accessibility of our approach.

**Figure 2:**
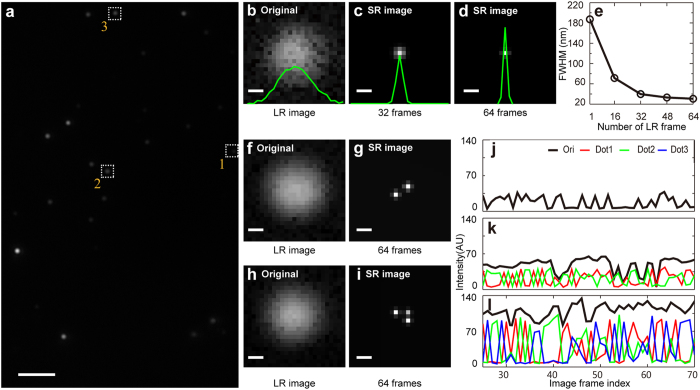
Test on quantum dots. (**a**) A single frame of QDs (diameter: 16 nm) from a series of LR images taken with translation between frames. (**b**) A close-up LR image of region 1 containing a bright signal corresponding to a single QD, where the green curve is the intensity profile in the horizontal direction. (**c**,**d**) Restored SR images using 32 and 64 LR observations respectively, with overlaid intensity profiles. (**e**) The observed FWHM of the restored quantum dot versus the number of LR observations. (**f**,**g**) Close-up LR and SR images of region 2 in **a**, where two QDs are resolved. (**h**,**i**) Close-up LR and SR images of region 3 in **a**, which show 3 QDs. (**j**) Intensity fluctuations over time in region 1 between bright and dark states (**k**) Intensity fluctuations of region 2, which are the sum of the intensities of the two resolved QDs in the SR image. (**l**) Intensity fluctuations of in region 3, which are made of the sum of the intensities of the three resolved QDs in the SR image. Scale bars 3 μm for (**a**) and 100 nm for (**b**–**d**) and (**f**–**i**)

We next examined quantum dots (QDs) acquired with excitation at 405 nm wavelength on a widefield microscope equipped with a 150 × 1.45 NA objective. This gives the diffraction limit 228 nm. A set of low resolution (LR) images were acquired whilst translating the sample in steps of 100 nm. Figure 2a shows a LR image containing several bright spots, with measured noise levels of σ_n_ = 11.2 (STD for 8-bit images). Figure 2b shows a zoom image of region 1, where the intensity profile is indeed Airy-disk shape of the FWHM of 194 nm (Gaussian fitting), in agreement with the theoretical value. Figure 2c,d shows restored SR images resulting from 32 and 64 LR observations, giving measured FWHM of 39.7 and 30.6 nm respectively; an exponential decrease on increasing LR frames is observed as shown in Fig. 2e, showing a resolution improvement of ~3-fold for 16 observations and up to 7-fold for 64 observations (this data was also shown in Fig. 1d for comparison). TRAM can indeed identify adjacent diffraction-unresolved multiple QDs. For example, for regions 2 and 3 of Fig. 2a (magnified in Fig. 2f,h), the single diffraction unresolved spot in fact contains 2 and 3 adjacent QDs respectively as shown in the restored images (Fig. 2g,i). To verify the results, we investigated QD intensity fluctuations, taking advantage of the quantum blinking effect of single QDs^17^. If a bright spot in the LR image contains a single dot, its intensity varies quantally between bright and dark states, as shown in Fig. 2j. However, if a spot contains two QDs, the signal is the sum of those of the two dots, consequently the “off” state appears less frequently (Fig. 2k). This characteristic becomes more prominent when there are more QD signals in a spot. Figure 2l shows the case of three QDs, where the intensity fluctuation tends to be averaged out by random blinks of all the individual dots in the region. Thus, deconvolving the intensity fluctuations over time alongside our image restoration provides a ‘ground truth’ for TRAM: our restoration can indeed separate single particles from diffraction-unresolved data.

**Figure 3:**
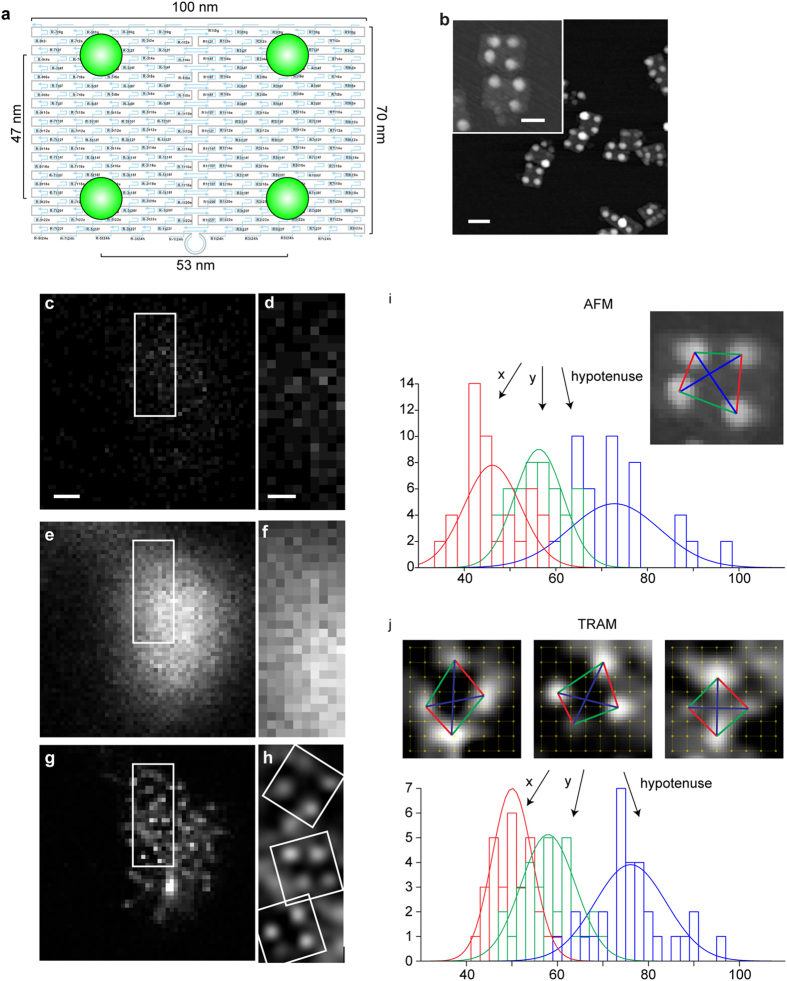
‘Ground-truth’ samples demonstrate the high-fidelity of TRAM. (**a**) Schematic of rectangular quantum dot structure on a DNA origami template. (**b**) AFM image of the quantum dot structure (scale bar 100 nm), where the inset shows a zoom area (scale bar 50 nm), confirming that our design is reiterated in the nano-structure. (**c**) A single diffraction-limited fluorescence image, from the 45 translation sequence, acquired using CLSM. Scale bar 100 nm. (**d**) A zoom region of interest from (**c**) Scale bar 50 nm. (**e**) Sum of all data from 45 aligned translation images, showing no structures in the over sampled data. (**f**) A zoom region of interest from (**e**). (**g**) TRAM super-resolution reveals rectangular structures in restored image. (**h**) A rendered, zoom region of interest from (**g**). (**i**) Measurement of the edges and hypotenuse of the structures from the AFM: (x) 46 nm, (y) 53 nm and (hypotenuse) 71 nm. (**j**) Similar measurements for TRAM: 50 nm, 58 nm and 76 nm, where three representative structures are shown in the pixel grid of 19 nm.

The so-called ‘ground truth’ is difficult to obtain in super-resolution imaging experiments, particularly if organic samples are required^16^. To further validate TRAM and confirm that our restoration approach delivered images that faithfully represent the original sample under observation, we designed and synthesized quantum-dot-DNA-origami structures of predictable dimensions. Quantum dots were again selected as fluorescent emitters for their predictable size and photo-stability, as we note that previously described organic dye-DNA origami^16^ is prone to photobleaching and could not be used in TRAM or in STED reliably. Each rectangular quantum dot structure was designed to measure 47 nm x 53 nm on a DNA origami template of 70 nm x 100 nm, as depicted in Fig. 3a. These structures were imaged using AFM to confirm their integrity (Fig. 3b); although the structures were designed to have four 16 nm-diameter quantum dots occupying each corner of the DNA origami scaffold, complete occupation of each conjugation site was difficult to achieve whilst avoiding over-saturation and non-conjugated quantum dot contamination. Nevertheless, this approach delivered sub-resolution samples, which although inevitably heterogeneous, generally had 3-4 quantum dots separated by a measureable and predictable distance distribution, confirmed using AFM (Fig. 3b, zoom in the inset). We next applied TRAM to this sample, prepared in an identical manner, acquiring 45 images translated with steps of ~100 nm in an X-Y grid-pattern on a CLSM with a 19 nm pixel size. Figure 3c shows a single diffraction-limited fluorescent image acquired using CLSM. As expected, no sub-diffraction structures can be seen in this image or in the zoom region of interest (Fig. 3d). By translating the sample many times (i.e. oversampling), more data are acquired, but a simple sum of these 45 (translation-corrected) images reveals no useful contrast (Fig. 3e,f). Importantly, however, these images provide useful information for TRAM restoration. A restored image from the same field of view is shown in Fig. 3g,h (rendered view). Distinct rectangular nano-structures are apparent in the TRAM image, reiterating the AFM data presented in Fig. 3b. To quantify this and take into account the heterogeneity of the samples, we measured the lengths of two edges (x and y) and hypotenuse of ~40 structures from both the AFM and TRAM images. The mean values found were (x) 46 nm, (y) 53 nm and (hypotenuse) 71 nm for AFM (Fig. 3i), the overlapping of the three histograms and their broad distributions are the manifestation of the structures imperfections as discussed above (Fig. 3b). We took the same measurement for TRAM, which is shown in Fig. 3j. To investigate the results further, we plot the three representative examples of structures that we have observed in the pixel grids of the image (19 nm), which can explain well the x, y and hypotenuse distributions in the histograms. For example, the x side mainly falls between 42 nm and 54 nm, which correspond to the diagonal lines of 1 by 2 pixels and 2 by 2 pixel, respectively. The mean values measured for TRAM were (x) 50 nm, (y) 58 nm and (hypotenuse) 76 nm (Fig. 3j), which describe rectangles of the same aspect ratio but is larger than the predicted and AFM values by a small fraction of the pixel size. The results are pleasing given the differences in sample preparation and imaging modality between AFM and TRAM. The TRAM restoration delivered, from data acquired using a standard confocal microscope, a very substantial improvement in spatial resolution and contrast, revealing sub-resolution rectangular structures with the shape we designed.

**Figure 4:**
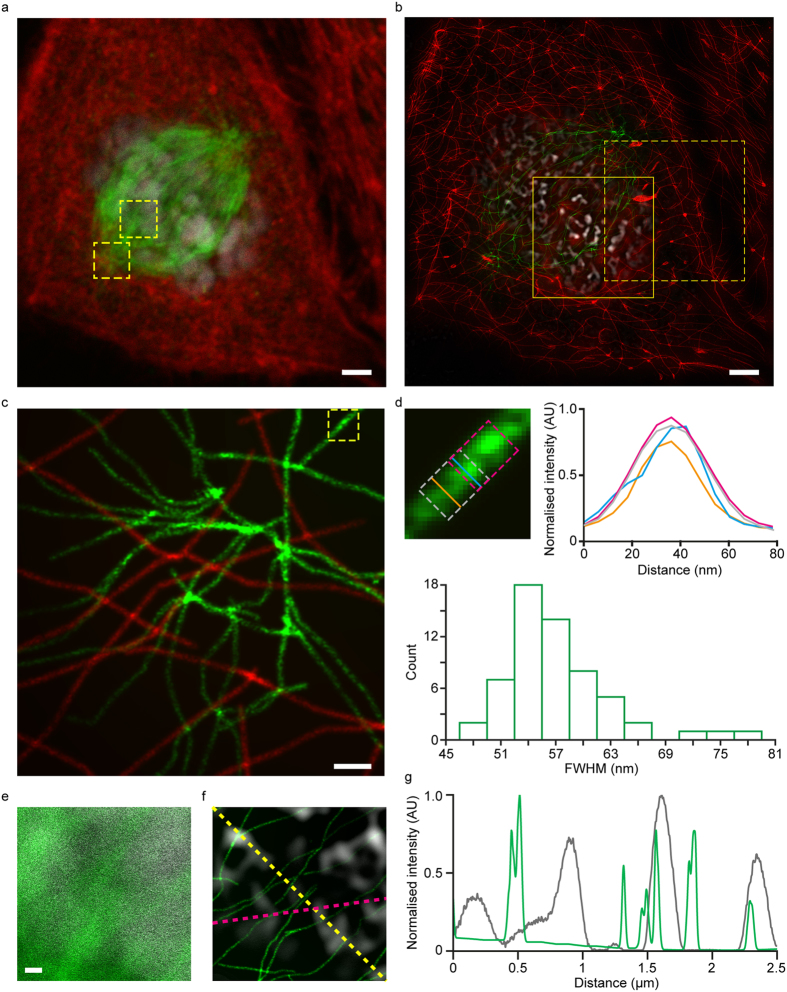
Multi-color TRAM of a pulmonary endothelial cell. (**a**) One of the 60 LR images acquired whilst translating the sample in XY-steps of ~100 nm. Three colors represent three different stained structures; red: actin, green: microtubules and gray: DNA (DAPI), respectively. Scale bar 2 μm. (**b**) TRAM-restored image using the 60 diffraction-limited images. (**c**) A zoom-view of the spindle-pole region (*bottom-left* box in (**a**)) showing TRAM restored data for both channels. Scale bar 250 nm. **(d)** Top row: A zoom-view of microtubule segment from the box in (**c**) and corresponding intensity profiles of the cross-section measurements at single pixel and within a window of 25 pixels respectively. Bottom row: Histogram of the FWHM measurements of 60 microtubule cross-sections, exhibiting a distinct peak at around 55 nm. (**e**) A zoom-view of microtubules (green) and DAPI stained nuclei (gray) from the top-right region-of-interest in (**a**). Scale bar 250 nm. (**f**) Corresponding TRAM restored region in which DAPI-stained structures are clearly resolved. (**g**) Intensity profiles of the microtubules (yellow line) and DAPI (pink line) from the TRAM restored image.

Having demonstrated that TRAM could deliver super-resolution images in a straightforward manner from relatively sparse samples, not requiring special instrumentation, we finally set out to acquire data from a biological sample. A principal requirement of biological imaging is multi-color labeling; however it remains challenging to acquire multi-color super-resolution image data. STED^2^ for example, commonly utilizes a single depletion laser, meaning that special combinations of organic dyes are required. Dual color photo-activated localization microscopy (PALM) experiments^5^ remain challenging until better green photo-activatable proteins become available. Multi-color direct stochastic optical reconstruction microscopy (dSTORM)^6^ also requires special sample preparation steps. In principle, TRAM is inherently multi-color, with no special sample preparation methodology required. We therefore selected a commercially available three-color sample, a bovine pulmonary artery endothelial cell sample stained with Texas Red-X phalloidin, anti-bovine α-tubulin detected with a BODIPY FL labeled secondary antibody, and DAPI (DAPI is shown in grayscale for clarity), to test multi-color TRAM; these commonly used dyes are not known for their special properties or particular photo-stability. A set of 60 diffraction-limited observations of all three channels was acquired in ~30 s, with translation steps in an XY-grid-pattern of ~100 nm between each frame, using CLSM. A single observation and its corresponding TRAM image are shown in Fig. 4a,b, respectively, with the latter demonstrating a large apparent improvement in resolution. A region corresponding to the centrosome is shown enlarged (Fig. 4c) highlighting the close proximity of actin (red) and microtubule (green) filaments in this region. To analyze the improvement in resolution in this multicolor image, we measure the FWHM of the cross sections of microtubule and actin filaments. Figure 4d (top row) shows a zoom microtubule segment with 4 measurements: 2 intersected by single pixel at slightly different locations and 2 averaged over a sliding window of 25 pixels. As seen, due to intensity fluctuations, the shape and width of the two cross sections vary considerably for the single pixel measurements but are almost unchanged when averaged over the 25 pixels. We therefore took the averaged measurements on 60 typical straight microtubule segments from different locations of the entire image. The histogram of the FWHM is shown in the bottom row of Fig. 4d, in which the distinct peak occurs at around 55 nm. The result is in a good agreement with the range of 50-60 nm observed by Gustafsson, Sedat and coworkers on their 3D SIM experiment^18^. We undertook a similar measurement to the actin network, finding the FWHM of the cross section to be around 80 nm. We further show a zoom view of microtubules and DAPI-stained nuclei in the CLSM and TRAM images (Fig. 4e,f) and TRAM restored super-resolution signal intensity profiles (Fig. 4g). As seen, microtubules has the FWHM around 55 nm, as discussed earlier, while the DAPI-stained nuclei is measured to be ~180 nm. The latter is larger than that measured using 3D SIM by Sedat and co-workers and probably shows that the current version of TRAM does not resolve blob-like structures as well as SIM.

We note that as with all other microscopy techniques, the different levels of structure and detail that TRAM can restore depend on its operating conditions. As biological samples have structures of more than one spatial scale and of different signal strengths, different parameter settings may be used in order to observe different levels of detail. As shown in Supplementary Fig. S4, for example, by varying the parameters controlling the denoising strength TRAM can reveal different details and structures, including actin bundles.

## Discussion

It is important to note that as with all other techniques, TRAM has limitations in its performance. It relies on intensity information at pixel level, so when bleaching occurs in a local region of an image, restoration is not possible there, but it does not affect other regions in the image. When there are changes in image structures between the translation images, either due to motion induced blur or dynamics, TRAM has difficulty in its present form and so is best suited to fixed samples (in common with many of the current super-resolution modalities). TRAM restoration currently requires a few hours of computation for a 256 × 256 image by using a desktop computer running on MATLAB, however, its performance will improve with algorithmic optimization, processing parallelization and with computing hardware advances. Moreover, as with other super-resolution microscopy techniques, including STED, a TRAM image depends on its operating conditions and, to a lesser extent than the existing techniques, sample preparation.

In summary, we present a super-resolution imaging technique, which can be used with any microscope without any hardware modification. TRAM is developed purposely to restore images under low SNR environments and containing small structures many times below the diffraction limit. Use of multiple transition observations in TRAM has enabled us to undertake more effective collaborative filtering for image denoising through a newly developed prior model. The multiple translation approach further imposes more stringent criteria on the SR restoration process; our restored image is collectively an optimal SR counterpart of all the diffraction-limited translation observations. This methodology has made TRAM more robust than the present SR methods when applied to fluorescence biological imaging. Our approach is multi-color, requires no special sample preparation, works with any fluorescent stain and is simple to apply, delivering super-resolution images with up to 7-fold improvement in lateral resolution. We believe that this technique will be of broad interest to the cell-biology community.

## Methods

### Super-resolution restoration method.

A low-resolution (LR) image, ***J***_*l*_, can be considered as the outcome of an original high-resolution (HR) image, ***I***_*l*_, after an image-degrading process involving blurring and noise contamination. This process can be formulated by a linear image capturing model^10^,


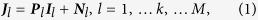


where *M* denotes the number of images (observations), the column vectors ***J***_*l*_ and ***I***_*l*_ consist of row-wise concatenations of the LR and HR images, ***P***_*l*_ is a blurring matrix determined by the PSF of the imaging system and ***N***_*l*_ represents noise contamination. The blurring matrix and noise can be different for different *l* in Eq.(1). SR restoration aims to recover the HR images beyond the diffraction limit from the diffraction-limited LR observations. Since the blurring matrix is usually not invertible^10^, the HR image is commonly estimated by minimizing a pre-defined energy function,


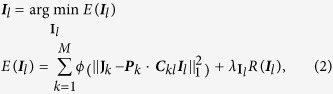


where the first term in the energy function, *E*(***I***_*l*_), measures the difference between the LR observations and predicted data in a l^2^-norm form, ***C***_*kl*_ is a shift matrix measuring the pixel-level correspondence between the HR images, ***I***_*l*_ and ***I***_*k*_, and 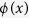 takes a robust function in the form of


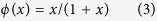


so that the energy function more likely reaches a global minimum^19^. In practice, the correspondence matrix***C***_*kl*_ is unknown to the observer but is assumed to be unchanged during the degrading process. As such, the matrix can be determined by the correspondence between LR images^11^. In the presence of noise, a prior model, *R*(***I***_*l*_), is essential to be included in the energy function to regularize the minimization process. The proportional parameter, 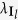, is to balance noise removal and feature preservation via the regularization. Since an edge is a fundamental feature that underlies more complicated features or structures in an image, many SR restoration methods in medical imaging have applied gradient operators (first-order difference) to build the prior model and achieved impressive performances in X-Ray mammography, fMRI and PET, as discussed earlier^9^. However, the gradient-based model does not work well when applied to biological fluorescence microscopy. Biological microscopy data are usually made up by vesicles, filaments, microtubules and their complex networks, which are more complicated than medical data that are usually images of organs or tissues. Spatial scales of the structures in the two types of images are also very different^9^,^12^. Since edges embedded in small and complex structures are prone to noise contamination, the gradient-based operators may not be able to robustly detect edges under noise contamination and therefore can fail to characterize and preserve complex structures. In a recent study^20^, we developed an anisotropic diffusion model by employing first- and second-order nonlocal differences (NLD) as feature detectors and demonstrated a superior performance of image restoration compared to many state-of-the-art denoising algorithms, particularly in high noise levels. This idea is based on our observations that diverse biological structures such as vesicles, filaments, microtubules are made primarily of two basic features, blob and ridge^21^,^22^, which are better characterized by a combination of these two NLDs.

In the present work, we further develop this idea by capturing and preserving spatially more complex high-order structures. This is necessary as SR restoration is a more complex operation that not only removes noise but also corrects structure distortion induced by blurring. To implement the idea, we associate each image pixel with a small spatial region (patch) around which it is centered. Pixels are then grouped based on local structure similarity of their corresponding patches by using un-supervised clustering methods^23^. A set of principal components (eigenvectors) for each group are then calculated using singular vector decomposition. Nonlocal features at various levels (orders) of complexity for each group are extracted by projecting the patches to their corresponding eigenvectors. These nonlinear features can be considered as a generalization of the first- and second-order NLDs used in our previous study to high orders and are adopted to build the prior model, *R*(***I***_*l*_), in Eq. (2) for SR restoration. The detail about the model is presented in supplementary text. We note that these nonlinear features, particularly those underlying fine structures, are prone to noise contamination. Making use of group statistics of similar patches has shown to improve the robustness of feature extraction^24^.

We solve the minimization problem in Eq. (2) by an iteratively reweighted least squares (IRLS) method, the flow chart of which is given in Supplementary Fig. S5. It has been proven in many studies^25^ that the IRLS leads to either the global optimum solution or a local optimum solution that is most close to the global optimum solution among all local optimum solutions. To solve Eq. (2), we assume the initial solution as ***I***_*l*_ = ***J***_*l*_. The solution then evolves iteratively while the energy function is gradually minimized by IRLS. The rate of the evolution is adjusted at each iteration step based on the difference of HR solutions between the present and previous steps. The parameter 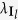 is also updated at each iteration step according to the residual noise contained in the current HR image estimation. When the difference of the HR image estimations between two adjacent iterations is below a pre-set threshold, the iteration stops and the solution is considered to the restored HR image. More details are given in the supplementary text.

### Translation microscopy (TRAM)

Based on the information theory^26^, the LR observations to be used to recover a HR image via the proposed inverse process must be correlated but not identical. For biological microscopy applications, the easiest way to obtain a set of (correlated) LR images is to record these images while the microscope or specimen is translated in the XY plane. The correspondence matrix in this case can be easily determined from motion vectors of the two LR images given by the relative positions between the camera and specimen^11^. Such multiple images were acquired in our lab by using a motorized stage translating in the X and the Y plane; the translation is calibrated at pixel level using image registration methods to avoid uncertainty due to any mechanic drifting. For example, 64 frames used in Fig. 4 were recorded at a frame rate of 498 ms in an 8 by 8 array in the XY plane. The distance between neighboring frames was set to be around or larger than the FWHM of PSF of the microscope. The 3D PSF is considered to be space variant^27^,^28^,^29^ and can be modelled based on experimental configurations^30^ or measured experimentally using point-like specimen such as beads and quantum dots. The 2D PSF in our TRAM restoration is the projection of the 3D PSF model^31^, taking into account the translation of each observation. We refer to the combination of such multiple LR image acquisition modality with our SR restoration method as translation microscopy (TRAM). Compared with other SR imaging techniques such as SIM, STED, dSTORM, etc., TRAM can be implemented simply on conventional microscopes; confocal, widefield and other imaging systems with little or no hardware modifications. We note that if optical sectioning techniques can acquires 3D diffraction-limited images with the depth (axial) information, TRAM can in principle be extended to be a 3D restoration method from the current 2D technique.

### Simulation

We applied our TRAM restoration method to a number of data sets and validated the results by comparing to the ground truths. We first tested our method on a 2-D standard resolution chart and a synthetic biological image containing blobs and ridges of varying sizes and orientations, from which we explore some usual scaling laws between the quality of restoration and number of LR images in different noise levels. Finally, we performed TRAM on two optical microscopy data sets, beads and quantum dots, with known ground truth. In all the tests the SR restoration process was measured by the mean squared difference-norm (MSDN) of the restored images between two adjacent iterations. When the MSDN reaches to a given threshold, the process was terminated and the solution is considered to be the HR image.

### Validation

#### 2-D standard resolution chart

An 8-bit HR resolution chart contains various features with varying sizes and orientations and is commonly used for evaluation of image restoration methods^32^, particularly improvement in SNR. We applied our TRAM method with a set of 64 LR images corrupted by a Gaussian-shaped PSF of Std *σ*_psf_ = 5, 10, 15 (pixels) and AWGN contamination of Std *σ*_n_ = 20 (Supplementary Fig. S1a for one of the observations and Supplementary Fig. S1b restored). The performance of TRAM is shown to improve monotonically on increasing the number of LR observations and begins to saturate at 50 LR images (Supplementary Fig. S1c), the saturation point is dependent on the noise level in the LR observations. We present the restored images and measurements by our method and other three popular SR methods, ZMT^33^, RSR^34^ and ALG^32^, in Supplementary Fig. S1d-j, the last of which is considered to be the state of art. By comparison, our results give the best visual and quantitative performance, restoring all the features in the original chart without artifacts.

#### Synthetic biological cell image

Pertinent to biological applications, we conducted the experiment on a synthetic cell image that contains blobs and ridges which mimic the transport particles and microtubules in intracellular structures (Supplementary Fig. S2a). The LR images were obtained by convolving Gaussian-shaped PSF of Std *σ*_psf_ = 31 pixels and noise of Std *σ*_n_ = 20 (Supplementary Fig. S2b). By comparing to the original HR image, the results show visually a remarkable resolution improvement, recovering all the original structures. The ratio of the averaged FWHM of the blobs and ridges in the restored image to that in the LR image is 6.3 (Supplementary Fig. S2d), which is a common measurement of resolution improvement in biology. We further investigated the resolution improvement of our method on different noise levels. In general, the FWHM ratio decreases as the noise level increase (Supplementary Fig. S2e). We finally studied the scaling of resolution improvement to the numbers of LR observations in different levels of noise contamination. In general, the FWHM ratio increases monotonically on increasing the LR number, as in the test of the standard resolution chart. However, higher noise reduces the maximum resolution that can be restored and also requires more LR observations for a same resolution improvement compared to lower noise case (Supplementary Fig. S2f). Note that the above restoration used the known PSF profile but did not require noise information; the restoration is robust under small fluctuations of the PSF.

### Software

We have implemented the TRAM algorithm in Matlab (Mathworks). The software has been attached here for demonstration purpose, which includes the TRAM program and a set of translation LR beads images. Users can run the program to show the evolution of how a TRAM SR image is restored from these LR images. The results can be compared with gSTED image from the same view area. The software can run in Windows7 64 bit and Linux Ubuntu 64 bit machines.

### Sample preparation

QDot 625 (Invitrogen) was diluted 1:1,000,000 in phosphate buffered saline (PBS). Coverslips were coated with CellTak (BD Biosciences) according to the manufacturer’s instructions. Diluted quantum dots were incubated on the coated coverslips for 1 hour prior to imaging in PBS. Fixed cell datasets were acquired using the FluoCells pre-prepared slide #2 (Invitrogen) which contains bovine pulmonary artery endothelial cells (BPAEC) stained with Texas Red-X phalloidin, anti-bovine α-tubulin and BODIPY FL labelled secondary antibody, and DAPI.

#### Quantum dot – DNA origami

All short staple strands were purchased from Invitrogen (China) and used as received. M13mp18 single stranded DNA was purchased from New England Biolabs. Chemicals were purchased from Sigma-Aldrich. Quantum dots were purchased from Invitrogen (China).

#### Preparation of DNA origami ruler

The rectangular shaped DNA origami template was formed according to the literature^35^. A molar ratio of 1:10 between the long viral ssDNA and the short helper strands was used. DNA origami was assembled in 1 × TAE-Mg^2+^ buffer (Tris 40 mM; Acetic acid 20 mM; EDTA 2 mM and Magnesium acetate 12.5 mM; pH 8.0) by cooling slowly from 95 °C to room temperature. DNA origami was then filtered with Millipore’s 100 kDa MWCO centrifuge filter to remove extra DNA helper strands. The concentration of the DNA origami was estimated from the optical absorbance at 260 nm. Purified DNA origami was mixed with streptavidin-coated QDs in 1 × TAE-Mg^2+^ buffer at room temperature.

#### AFM characterization

The DNA origami ruler sample was deposited onto a new cleaved mica substrate. Then the surface was rinsed using deionized water and dried by air. All AFM images were obtained using a Multimode Nanoscope VIII instrument (Bruker) under tapping mode in air with NSC11 tips (μMasch).

### Fluorescent image data acquisition

Quantum dot calibration data were acquired on an inverted IX81 microscope (Olympus) using a 150 × 1.45 NA objective. Illumination was provided by a fully motorized four laser TIRF combiner coupled to a 405 nm 100 mW laser under widefield illumination. The sample was laterally translated using a motorized stage (ASI). Image data was collected using an Orca-Flash 4.0 s CMOS camera (Hamamatsu) which in combination with a 1.6 × magnifier in the image path provided an effective pixel size of 27 × 27 nm. Ten frames were acquired at each position before translation of the stage to the next position. Fixed cell and DNA origami image data were acquired on an SP5 gSTED SMD laser scanning confocal microscope (Leica) using a 60 × 1.4 NA objective. Images 4096 × 4096 were acquired with a pixel size of 6 nm (for Fig. 4) or 19 nm (for Fig. 3). A single frame in each channel was acquired before translation of the stage to the next position.

## Additional Information

**How to cite this article**: Qiu, Z. *et al.* Translation Microscopy (TRAM) for super-resolution imaging. *Sci. Rep.* **6**, 19993; doi: 10.1038/srep19993 (2016).

## Supplementary Material

Supplementary Information
