# Corrigendum: Translation Microscopy (TRAM) for super-resolution imaging

**DOI:** 10.1038/srep22313

**Published:** 2016-03-18

**Authors:** Zhen Qiu, Rhodri S. Wilson, Yuewei Liu, Alison R. Dun, Rebecca S. Saleeb, Dongsheng Liu, Colin Rickman, Margaret Frame, Rory R. Duncan, Weiping Lu

Scientific Reports 6: Article number: 1999310.1038/srep19993; published online: 01292016; updated: 03182016

In the original version of this Article, Yuewei Liu was incorrectly listed as being affiliated with 'Department of Chemistry, Tsinghua University, Beijing, China'. The correct affiliation is listed below:

Edinburgh Super-Resolution Imaging Consortium, Heriot-Watt University, Edinburgh, EH14 4AS.

In addition, the Supplementary Software was omitted.

These errors have now been corrected in the PDF and HTML versions of the Article

