# Supplementary material for: Translation Microscopy (TRAM) for super-resolution imaging
Source: Sci Rep. 2016 Jan 29;6:19993. doi: 10.1038/srep19993 (PMC4731806; doi:10.1038/srep19993)
Supplement: Supplementary Information [file srep19993-s1.pdf]

Translation Microscopy (TRAM) for super-resolution imaging.

Zhen Qiu*[1,2,3], Rhodri S Wilson*[1,3], Yuewei Liu[1,3], Alison Dun[1,3], Rebecca S Saleeb[1,3], Dongsheng Liu[4], Colin Rickman[1,3], Rory R Duncan[1,3,5], Weiping Lu[1,3,5]

*Authors contributed equally to the work.

[1] Institute of Biological Chemistry, Biophysics and Bioengineering, Heriot-Watt University, Edinburgh, EH14 4AS, [2] Present address: Centre for Neuroregeneration Chancellor's Building 49 Little France Crescent Edinburgh EH16 4SB, [3] Edinburgh Super-Resolution Imaging Consortium, www.esric.org. [4]Department of Chemistry, Tsinghua University, Beijing, China

[5] To whom correspondence should be addressed; email: W.Lu@hw.ac.uk, Telephone: +44(0)131 451 3065 or R.R.Duncan@hw.ac.uk, telephone: +44 (0)131 451 3414

Supplementary Notes

1. Method of TRAM

The full flow chart for the method of TRAM can be found in Supplementary Figure 5. Here we discuss in more detail each of the steps in the TRAM restoration process.

1.1 Energy function

Following the general form of Eq. (2) in **Methods,** we define the energy function as

$$E(\boldsymbol{I}_l)=\sum_{k=1}^{M}\phi\left(\left\|\boldsymbol{J}_k - \boldsymbol{P}_k\cdot\boldsymbol{C}_{kl}\boldsymbol{I}_l\right\|_1^2\right)+\lambda_{I_l}\sum_{c_t=1}^{N_c}\sum_{x\in c_t}\phi\left(\sum_{h=1}^{O_c}w_h(x)\left(f^{(h)}\left(\boldsymbol{L}_x\right)\right)^2\right), \quad (1)$$

which comprises the first term measuring the difference between the low-resolution observations and predicted high-resolution counterpart and the second term characterizing image structures to regularize the minimization process.

In the second term, $\boldsymbol{L}_x$ represents a patch of 2*W*+1 pixels centred around *x* and is a vector comprising intensities of all pixels, i.e.,

$$\boldsymbol{L}_x=[I(x-W),I(x-w+1),\dots I(x),\dots I(x+W)]^T . \quad (2)$$

The function $f^{(h)}\left(\boldsymbol{L}_x\right)$ is the hth principal component (PC) score, which is the projection of the patch $\boldsymbol{L}_x$ onto a low-dimensional space using principle component analysis (PCA). PCA has been widely used in computer vision automatic feature extraction from complex data sets [1]. Here, $f^{(h)}\left(\boldsymbol{L}_x\right)$ characterizes nonlocal features of images through analysis of patches and can be considered as a generalization of the first- and second-order nonlocal differences (NLDs) in our previous study. For example, $f^{(1)}\left(\boldsymbol{L}_x\right)$ for patches containing an edge feature is equivalent to the 1st-order NLD, while $f^{(2)}\left(\boldsymbol{L}_x\right)$ for a ridge/blob feature represents the 2nd-order NLD.

As discussed in **Methods**, nonlinear features characterized by $f^{(h)}\left(\boldsymbol{L}_x\right)$, particularly those underlying fine structures are prone to noise contamination. It is therefore advantageous to group and characterize patches of similar structures in order to enhance the robustness of feature extraction [2]. We apply a traditional K-means clustering algorithm [3] to the image patches, $\left\{\boldsymbol{L}_x\right\}_{x=1,\dots N}$, to partition all pixels $\left\{x\in ¥^+ \middle| x\le N\right\}$ into N_c separate groups $\left\{c_t\right\}_{t=1,\dots,N_c}$, under the conditions [3],

$$\begin{cases} c_t \neq Null \\ \cup_{t=1}^{N_c} c_t = \left\{ x \in ¥^{+} \middle| x \leq N \right\} \\ c_p \cap c_q = Null,\ p \neq q \end{cases}. \tag{3}$$

Given the clustering result in Eq.(3), PC score $f^{(h)}\left(\boldsymbol{L}_x\right)$ is estimated for pixel *x* belonging to each patch group by using PCA as

$$\begin{aligned} &\boldsymbol{X} = [\boldsymbol{L}_1, \cdots, \boldsymbol{L}_x, \cdots] = \boldsymbol{U} \sum \boldsymbol{V}^T, \quad x \in c_t \\ &\boldsymbol{V} = \left[\boldsymbol{v}_0^T, \boldsymbol{v}_1^T, \boldsymbol{v}_h, \ldots, \boldsymbol{v}_{2W+1}^T \right]^T \\ &\boldsymbol{\beta}_x = \left[f^{(0)}(\boldsymbol{L}_x), \ldots, f^{(h)}(\boldsymbol{L}_x), \ldots \right]^T = \boldsymbol{V}\boldsymbol{L}_x \end{aligned}, \tag{4}$$

where $\boldsymbol{V}$ is a matrix whose columns are made by eigenvectors $\boldsymbol{v}_h$ of the matrix $\boldsymbol{X}^T\boldsymbol{X}$, $\boldsymbol{\beta}_x$ is a column vector whose h^{th} element is $f^{(h)}(\boldsymbol{L}_x)$. The summation in the second term of Eq. (1) is therefore applied first to patches in a group of similar features and then to all different groups.

The number of PC scores used for feature modeling, O_{c_t}, in Eq. (1) is critical in super-resolution restoration, because a small number may miss key structures, while a large number can result in over-fitting, namely turning noise into features. To overcome the problem, less informative $f^{(h)}\left(\boldsymbol{L}_x\right)$, which are often small in values, should be discarded. We apply a simple hard shrinkage method [4],

$$f^{(h)}(\boldsymbol{L}_x) = \begin{cases} f^{(h)}(\boldsymbol{L}_x) & f^{(h)}(\boldsymbol{L}_x) > \gamma_{c_t} \\ 0 & else \end{cases}. \tag{5}$$

where the threshold γ_{c_t} is determined by the noise level of the patches in group c_t. The highest order of the PC scores $f^{(h)}\left(\boldsymbol{L}_x\right)$ for all pixels $x \in c_t$ in the group is then estimated as the average number of non-zero elements among all vectors $\left\{\boldsymbol{\beta}_x\right\}_{x \in c_t}$, that is,

$$O_{c_t} = \frac{\sum_{x \in c_t} \left\| \boldsymbol{\beta}_x \right\|_0}{\left| C_t \right|}, \tag{6}$$

where the l^0-norm $\|\boldsymbol{\beta}_x\|_0$ of the vector $\boldsymbol{\beta}_x$ is the number of its non-zero elements and $|C_t|$ represents the number of pixels in the group c_t. The weighing coefficient $w_h(x)$ in Eq. (1) is define as, $w_h(x) = f^{(h)}(\boldsymbol{L}_x) \Big/ \sum_{r=1}^{O_{c_t}} f^{(r)}(\boldsymbol{L}_x)$, which enhances the effects of large scores.

1.2 Energy minimization

We minimize the energy function of Eq. (1), $\boldsymbol{I}_l = \arg\min_{\boldsymbol{I}_l} E(\boldsymbol{I}_l)$, to estimate the desired high-resolution image. This can be done by solving the gradient equation, $\frac{dE(\boldsymbol{I}_l)}{d\boldsymbol{I}_l} = 0$. We rewrite Eq. (1) in a matrix-vector form before differentiation. We first define a matrix, $\boldsymbol{D}_h \in \mathbf{R}^{N\times N}$, which corresponds to the hth PC score $f^{(h)}(\boldsymbol{L}_x)$ of all pixels (patches),

$$\boldsymbol{D}_h = \overbrace{\begin{bmatrix} \boldsymbol{v}_{W+1,h}^T & 0 & \cdots & 0 \\ 0 & \boldsymbol{v}_{W+2,h}^T & 0 & \cdots \\ \vdots & 0 & \ddots & \vdots \\ 0 & 0 & \cdots & \boldsymbol{v}_{N-W,h}^T \end{bmatrix}}^{N} \Bigg\} N \tag{7}$$

where $\boldsymbol{v}_{x,h}$ is the hth eigenvector defined by Eq. (4). We further define a column vector, $\boldsymbol{\delta}_x \in \mathbf{R}^{N\times 1}$, whose x^{th} element is the only nonzero element with unit value so that the xth element of any vector $v = [v(1),\ldots v(x),\ldots v(N)]^T$ can be written as

$$\boldsymbol{v}(x) = \boldsymbol{\delta}_x^T \boldsymbol{v}\,. \tag{8}$$

Combining Eq.(4), (7) and (8), we can rewrite the scalar variable of the PC score as

$$f^{(h)}(\boldsymbol{L}_x) = \boldsymbol{\delta}_x^T \boldsymbol{D}_h \boldsymbol{I}_l\,. \tag{9}$$

The function $E(\boldsymbol{I}_l)$ can then be expressed by using Eq. (9) in the following matrix-vector form,

$$E(\boldsymbol{I}_l) = \sum_{k=1}^{M}\sum_{x=1}^{N}\phi\left(\left[\boldsymbol{\delta}_x^T\left(\boldsymbol{P}_k \cdot \boldsymbol{C}_{kl}\boldsymbol{I}_l - \boldsymbol{J}_k\ \right)\right]^2\right)$$
$$+\lambda_{\boldsymbol{I}_l}\sum_{c_t=1}^{N_c}\sum_{x\in c_t}\phi\left(\sum_{h=1}^{O_{c_t}} w_h(x)\left(\boldsymbol{\delta}_x^T\boldsymbol{D}_h\boldsymbol{I}_l\right)^2\right) \quad (10)$$
,

which allows us to directly computer the gradient,

$$\frac{dE(\boldsymbol{I}_l)}{d\boldsymbol{I}_l} = \left(\lambda_{\boldsymbol{I}_l}\boldsymbol{A}_{PCS,l} + \lambda_{\boldsymbol{I}_l}\boldsymbol{S}_{PCS,l} + \sum_{k=1}^{M}\boldsymbol{C}_{kl}^T\boldsymbol{P}_k^{\ T}\boldsymbol{A}_k\boldsymbol{P}_k\boldsymbol{C}_{kl}\right)\boldsymbol{I}_l$$
$$-\sum_{k=1}^{M}\boldsymbol{C}_{kl}^T\boldsymbol{P}_k^{\ T}\boldsymbol{A}_k\boldsymbol{J}_k \quad (11)$$
,

where the N × N diagonal matrices, $\boldsymbol{A}_{PCS,l}$ and $\boldsymbol{S}_{PCS,l}$, are given as

$$\boldsymbol{A}_{PCS,l} = \sum_{c_t=1}^{N_c}\sum_{h=1}^{O_{c_t}}\sum_{x\in c_t} w_h(x)\phi'\left(\sum_{h=1}^{O_{c_t}} w_h(x)\left(\boldsymbol{\delta}_x^T\boldsymbol{D}_h\boldsymbol{I}_l\right)^2\right)\boldsymbol{D}_h^T\boldsymbol{\delta}_x\boldsymbol{\delta}_x^T\boldsymbol{D}_h ,$$
$$\boldsymbol{S}_{PCS,l} = \sum_{c_t=1}^{N_c}\sum_{h=1}^{O_{c_t}}\sum_{x\in c_t} w_h(x)\phi'\left(\sum_{h=1}^{O_{c_t}} w_h(x)\left(\boldsymbol{\delta}_x^T\boldsymbol{D}_h\boldsymbol{I}_l\right)^2\right)\boldsymbol{\delta}_x\boldsymbol{I}_l^T\frac{d\boldsymbol{D}_h}{d\boldsymbol{I}_l}\boldsymbol{\delta}_x^T\boldsymbol{D}_h \quad (12)$$

and the N × N diagonal matrix , $\boldsymbol{A}_k$, is given as

$$\boldsymbol{A}_k = \mathrm{diag}\left(\boldsymbol{\Phi}_k\right)$$
$$\boldsymbol{\Phi}_k = \left[\phi'\left(\left(\boldsymbol{\delta}_1^T\left(\boldsymbol{P}_k \cdot \boldsymbol{C}_{kl}\boldsymbol{I}_l - \boldsymbol{J}_k\right)\right)^2\right),\ldots\phi'\left(\left(\boldsymbol{\delta}_N^T\left(\boldsymbol{P}_k \cdot \boldsymbol{C}_{kl}\boldsymbol{I}_l - \boldsymbol{J}_k\right)\right)^2\right)\right]^T . \quad (13)$$

The minimization, i.e., $\frac{dE(\boldsymbol{I}_l)}{d\boldsymbol{I}_l} = 0$, leads to the following equation,

$$\left(\lambda_{\boldsymbol{I}_l}\boldsymbol{A}_{PCS,l} + \lambda_{\boldsymbol{I}_l}\boldsymbol{S}_{PCS,l} + \sum_{k=1}^{M}\boldsymbol{C}_{kl}^T\boldsymbol{P}_k^{\ T}\boldsymbol{A}_k\boldsymbol{P}_k\boldsymbol{C}_{kl}\right)\boldsymbol{I}_l = \sum_{k=1}^{M}\boldsymbol{C}_{kl}^T\boldsymbol{P}_k^{\ T}\boldsymbol{A}_k\boldsymbol{J}_k \quad (14)$$

which is a nonlinear equation of $\boldsymbol{I}_l$ because $\boldsymbol{A}_{PCS,l}$, $\boldsymbol{S}_{PCS,l}$ and $\boldsymbol{A}_k$ also involve the variable $\boldsymbol{I}_l$, so will have multiple solutions that correspond to local and global minima of the energy function $E(\boldsymbol{I}_l)$. We here apply a modified iterative reweighted least squares (MIRLS) method [5, 6] that

iteratively estimates between $\boldsymbol{A}_{PCS,l}$, $\boldsymbol{S}_{PCS,l}$, $\boldsymbol{A}_k$ and $\boldsymbol{I}_l$. To do so, we first fix $\boldsymbol{I}_l$, so from Eq.(12), $\frac{d\boldsymbol{D}_h}{d\boldsymbol{I}_l}=0$ and $\boldsymbol{S}_{PCS,l}$ goes to 0. We can then rewrite Eq. (14) as

$$\sum_{k=1}^{M}\left(\boldsymbol{B}_k-\boldsymbol{Q}_k\right)=\lambda_{\boldsymbol{I}_l}\boldsymbol{A}_{PCS,l}\boldsymbol{I}_l \quad (15)$$

,

where the matrices $\boldsymbol{B}_k$ and $\boldsymbol{Q}_k$ are given respectively as

$$\begin{aligned}\boldsymbol{B}_k &= \boldsymbol{C}_{kl}^T\boldsymbol{P}_k^T\boldsymbol{A}_k\boldsymbol{J}_k \\ \boldsymbol{Q}_k &= \boldsymbol{C}_{kl}^T\boldsymbol{P}_k^T\boldsymbol{A}_k\boldsymbol{P}_k\boldsymbol{C}_{kl}\boldsymbol{I}_l\end{aligned} . \quad (16)$$

We finally rewrite the nonlinear equation Eq. (15) as

$$\begin{cases}\boldsymbol{B}_1-\boldsymbol{Q}_1=\lambda_{\boldsymbol{I}_1}\boldsymbol{A}_{PCS,l}\boldsymbol{I}_l/M \\ \vdots \\ \boldsymbol{B}_k-\boldsymbol{Q}_k=\lambda_{\boldsymbol{I}_k}\boldsymbol{A}_{PCS,k}\boldsymbol{I}_l/M \\ \vdots \\ \boldsymbol{B}_M-\boldsymbol{Q}_M=\lambda_{\boldsymbol{I}_M}\boldsymbol{A}_{PCS,M}\boldsymbol{I}_l/M\end{cases} . \quad (17)$$

We note that Eq.(17) has more constraint than Eq. (15) since the unknown image $\boldsymbol{I}_l$ now must satisfy a set of *M* equations simultaneously, i.e., satisfy each of the *M* observations, instead the sum of the *M* observations. The solution of Eq. (17) will be more likely to get closer to the ground truth than that of Eq. (15).

1.3 Algorithm Pipelines

The main steps of MIRLS for solving Eq. (17) are:

(a) Initialization: Let $\boldsymbol{I}_l$ = $\boldsymbol{J}_l$ and $\lambda_{\boldsymbol{I}_l}$ = σ_n, where LR observation $\boldsymbol{J}_k$, the blurring matrix $\boldsymbol{P}_k$, the correspondence matrix $\boldsymbol{C}_{kl}$ and the noise Standard deviation σ_n are known.

(b) Computer the weight matrices $\boldsymbol{B}_k$, $\boldsymbol{A}_{PCS,k}$, $\boldsymbol{Q}_k$ by Eq. (12) and (16) based on the current estimate $\boldsymbol{I}_l$.

(c) For each observation *k*:

(c1) Solve the equation $\hat{\boldsymbol{I}}_{l,k} = \operatorname{argmin}\left\|\frac{1}{2}\boldsymbol{B}_k - \boldsymbol{Q}_k\right\|_2^2$ to obtain an intermediate solution $\hat{\boldsymbol{I}}_{l,k}$.

(c2) Use the above $\hat{\boldsymbol{I}}_{l,k}$ as the initial value to refine the solution by solving equation $\boldsymbol{I}_{l,k} = \arg\min\left\|\lambda_{\boldsymbol{I}_k}\boldsymbol{A}_{PCS,k}\hat{\boldsymbol{I}}_{l,k} / M - \frac{1}{2}\boldsymbol{B}_k\right\|_2$.

(d) The solution $\boldsymbol{I}_l$ is obtained by a weighted average of $\{\boldsymbol{I}_{l,k}\}_{k=1,\dots M}$

$$\boldsymbol{I}_l = \left[\sum_{k=1}^{M}\boldsymbol{w}_1(k)\boldsymbol{I}_{l,k}(1), \sum_{k=1}^{M}\boldsymbol{w}_2(k)\boldsymbol{I}_{l,k}(2), \dots \sum_{k=1}^{M}\boldsymbol{w}_N(k)\boldsymbol{I}_{l,k}(N)\right]^T \quad (18)$$

where the weight vector is given as

$$\boldsymbol{w}_i = \left[\phi'(\boldsymbol{I}_1(i) - \boldsymbol{I}_l(i)), \dots \phi'(\boldsymbol{I}_M(i) - \boldsymbol{I}_l(i))\right]^T / C_k, i = 1,2\dots,N \quad (19)$$

and ϕ' is the derivative of the robust function. C_k is a normalization factor. This step enforces that the multiple solutions $\boldsymbol{I}_{l,k}$ by step (c) should be similar to each other.

(e) Go to step (c) if Eq. (14) cannot be satisfied using the current estimation $\boldsymbol{I}_l$; otherwise update the parameter $\lambda_{\boldsymbol{I}_l}$ according to the residual noise in $\boldsymbol{I}_l$ [7].

(f) The iteration stops when $\boldsymbol{I}_l$ converges to a certain level, measured by MSDN between two adjacent images, and is considered to be the restored image; otherwise go to step (b) to compute again the weight matrices with an updated $\boldsymbol{I}_l$.

The intermediate solution $\hat{\boldsymbol{I}}_{l,k} = \operatorname{argmin}\left\|\frac{1}{2}\boldsymbol{B}_k - \boldsymbol{Q}_k\right\|_2^2$ in Step (c1) can be solved by many approaches, such as conjugate gradient (CG), Wiener Filter, or shrinkage method. We solve it by using an iterative Wiener Filter method with a slight modification [8] as

$$\hat{\boldsymbol{I}}_{l,k} = \frac{1}{2}\left(\boldsymbol{I}_{l,k}^{\text{pre}} + \left(\boldsymbol{P}_k^T\boldsymbol{P}_k + \text{eps}\right)^{-1}(\boldsymbol{P}_k^T\boldsymbol{J}_k - \boldsymbol{P}_k^T\boldsymbol{P}_k\boldsymbol{I}_{l,k}^{\text{pre}})\right) \quad (20)$$

where eps is a small constant to make sure the stability of the matrix inverse, $\boldsymbol{I}_{l,k}^{\text{pre}}$ is the solution for the previous iteration. Given the intermediate solution, $\hat{\boldsymbol{I}}_{l,k}$, we can then solve in step (c2) the equation

$$\boldsymbol{I}_{l,k} = \arg\min \left\| \lambda_{\boldsymbol{I}_k} \boldsymbol{A}_{PCS,k} \hat{\boldsymbol{I}}_{l,k} / M - \frac{1}{2} \boldsymbol{B}_k \right\|_2^2 \tag{21}$$

which is supposed to be also solved by Wiener filter but its stability cannot be guaranteed. We therefore revise the equation by adding a regularization term in Eq. (21) as

$$\boldsymbol{I}_{l,k} = \arg\min \left\| \lambda_{\boldsymbol{I}_k} \boldsymbol{A}_{PCS,k} \hat{\boldsymbol{I}}_{l,k} / M - \frac{1}{2} \boldsymbol{B}_k \right\|_2^2 + \left\| \left(\text{Root}(\boldsymbol{A}_{PCS,k}) \right)^{-1} \boldsymbol{I}_{l,k} \right\|_1 \tag{22}$$

where $\left\| \bullet \right\|_1$ is the l^1 norm and operator $\text{Root}\left(\boldsymbol{A}_{PCS,k} \right)$ indicates a new matrix whose elements are square root of the corresponding elements of the matrix $\boldsymbol{A}_{PCS,k}$. We can then solve Eq. (22) using a well-known method of least-absolute-shrinkage-and-selection-operator (lasso) [9] as,

$$\begin{aligned} \boldsymbol{I}_{l,k}(x) = {} & \delta_x \boldsymbol{D}_1 \text{Root}\left(\boldsymbol{A}_{PCS,k} \right)^{-1} \text{sgn}\left(\text{Root}\left(\boldsymbol{A}_{PCS,k} \right)^{-1} \boldsymbol{D}_1 \boldsymbol{I}_{l,k} \right) \\ & \times \left(\left| \text{Root}\left(\boldsymbol{A}_{PCS,k} \right)^{-1} \boldsymbol{D}_1 \boldsymbol{I}_{l,k} \right| - \lambda_{\boldsymbol{I}_k} / M \right)^{+} \end{aligned} \tag{23}$$

,

where $\text{sgn}\left(\bullet \right)$ is a sign function and $\left(\bullet \right)^{+}$ is a shrinkage function given as [6],

$$\left(x - a \right)^{+} = \begin{cases} x - a & \left| x - a \right| > 0 \\ 0 & else \end{cases} \tag{24}$$

References

1. Jolliffe, I. Principal component analysis. (Wiley Online Library, 2005).
2. Mairal, J., Bach, F., Ponce, J., Sapiro, G. & Zisserman, A. in Proc. CVPR 2009 2272-2279 (IEEE, 2009).
3. Jain, A.K., Murty, M.N. & Flynn, P.J. Data clustering: a review. *Acm Computing Surveys (CSUR)* **31**, 264-323 (1999).

4. Elad, M. Why simple shrinkage is still relevant for redundant representations? *IEEE Transactions on Information Theory* **52**, 5559-5569 (2006).
5. Daubechies, I., Defrise, M. & De Mol, C. An iterative thresholding algorithm for linear inverse problems with a sparsity constraint. *Communications on pure and applied mathematics* **57**, 1413-1457 (2004).
6. Daubechies, I., DeVore, R., Fornasier, M. & Güntürk, C.S.n. Iteratively reweighted least squares minimization for sparse recovery. *Commun. Pure Appl. Math.* **63**, 1-38 (2010).
7. Osher, S., Burger, M., Goldfarb, D., Xu, J. & Yin, W. An iterative regularization method for total variation-based image restoration. *Multiscale Modeling & Simulation* **4**, 460-489 (2005).
8. Hillery, A.D. & Chin, R.T. Iterative Wiener filters for image restoration. *Signal Processing, IEEE Transactions on* **39**, 1892-1899 (1991).
9. Tibshirani, R. Regression shrinkage and selection via the lasso. *Journal of the Royal Statistical Society. Series B (Methodological)*, 267-288 (1996).

Supplementary Figure S1

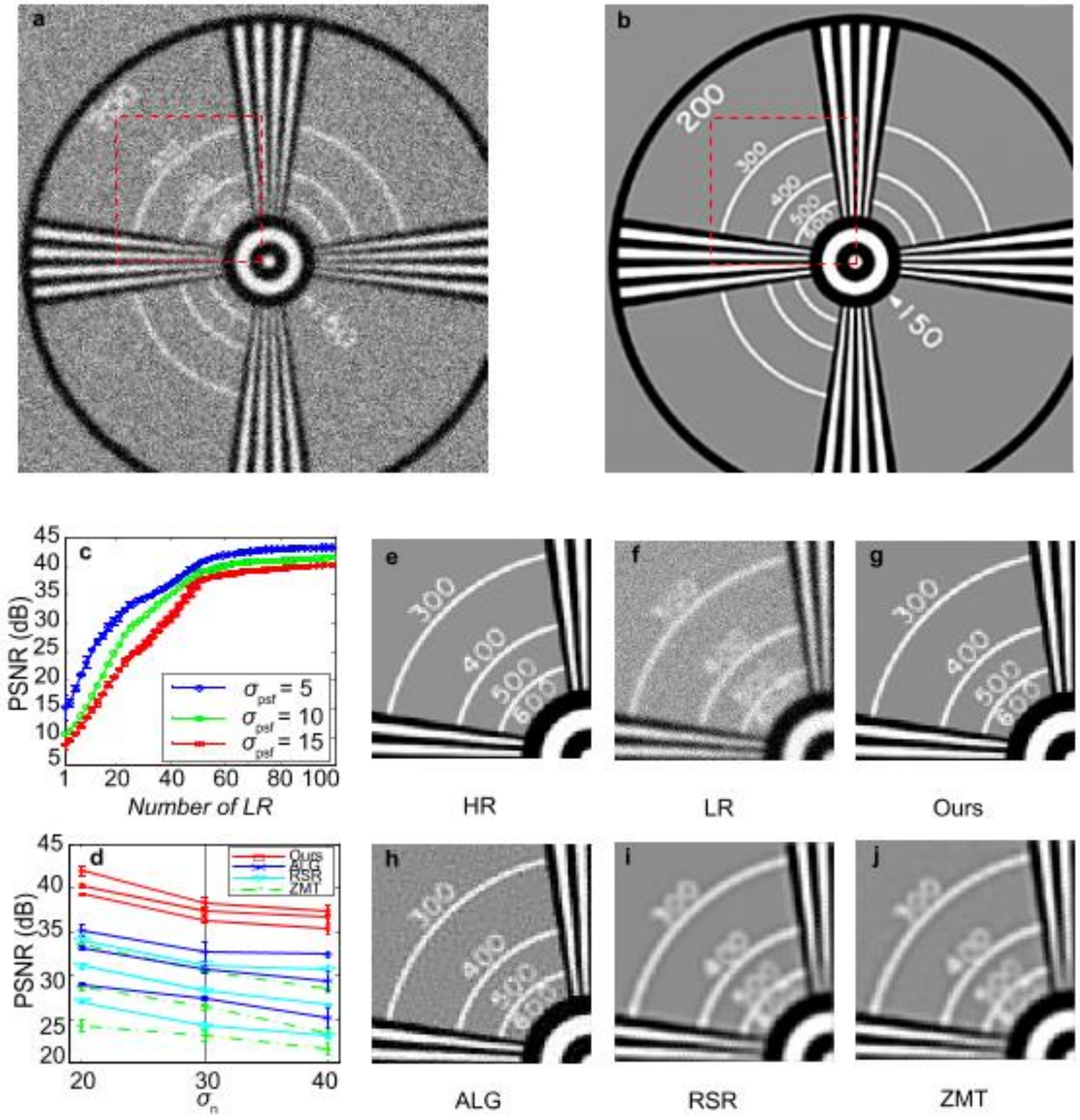

Supplementary Figure S2

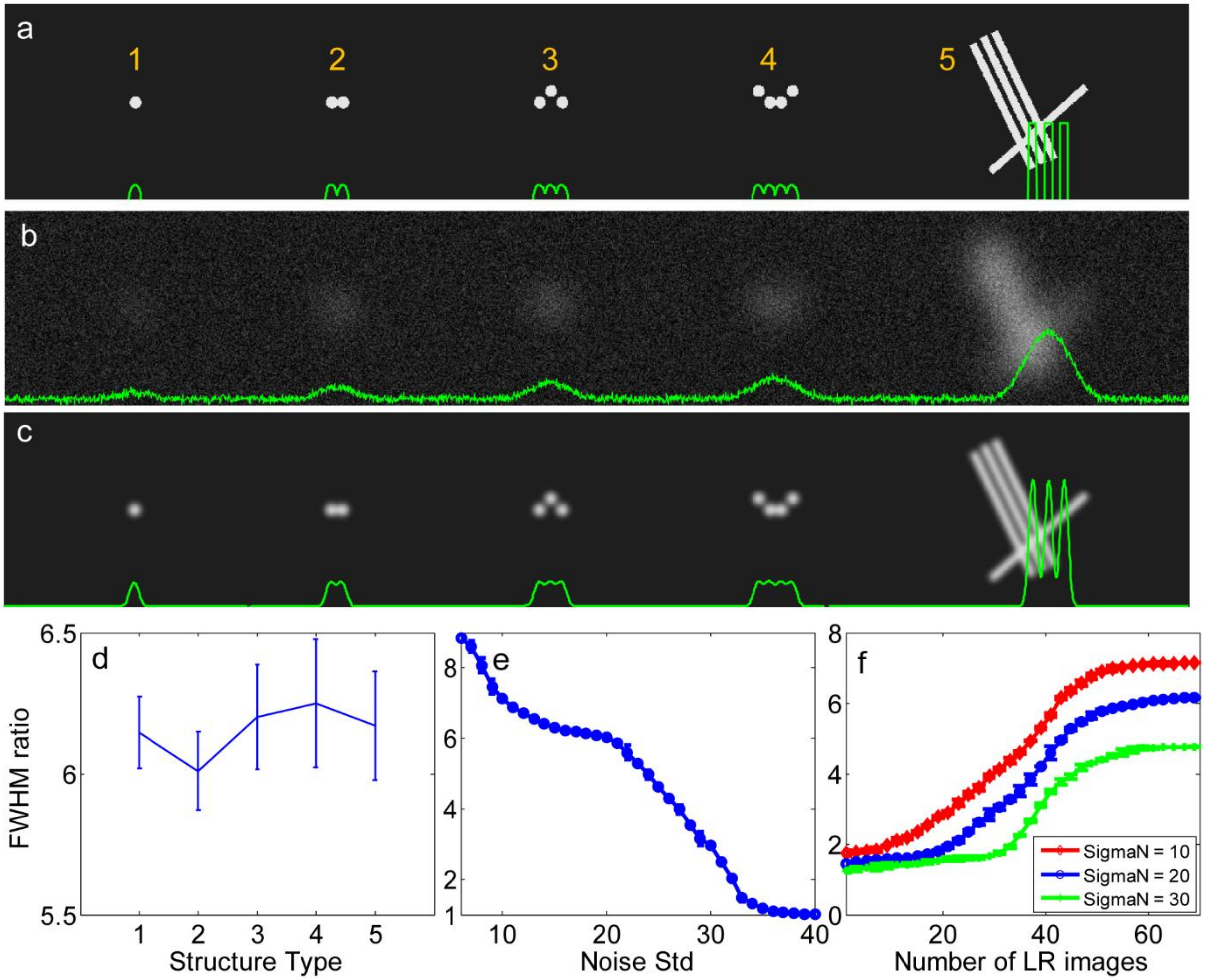

Supplementary Figure S3

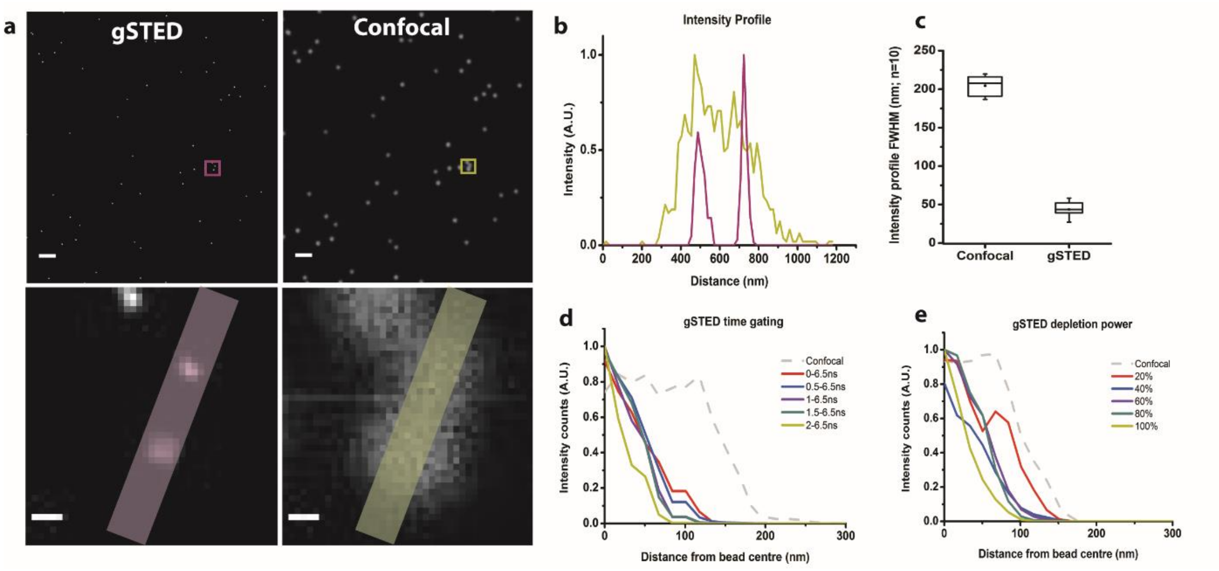

Supplementary Figure S4

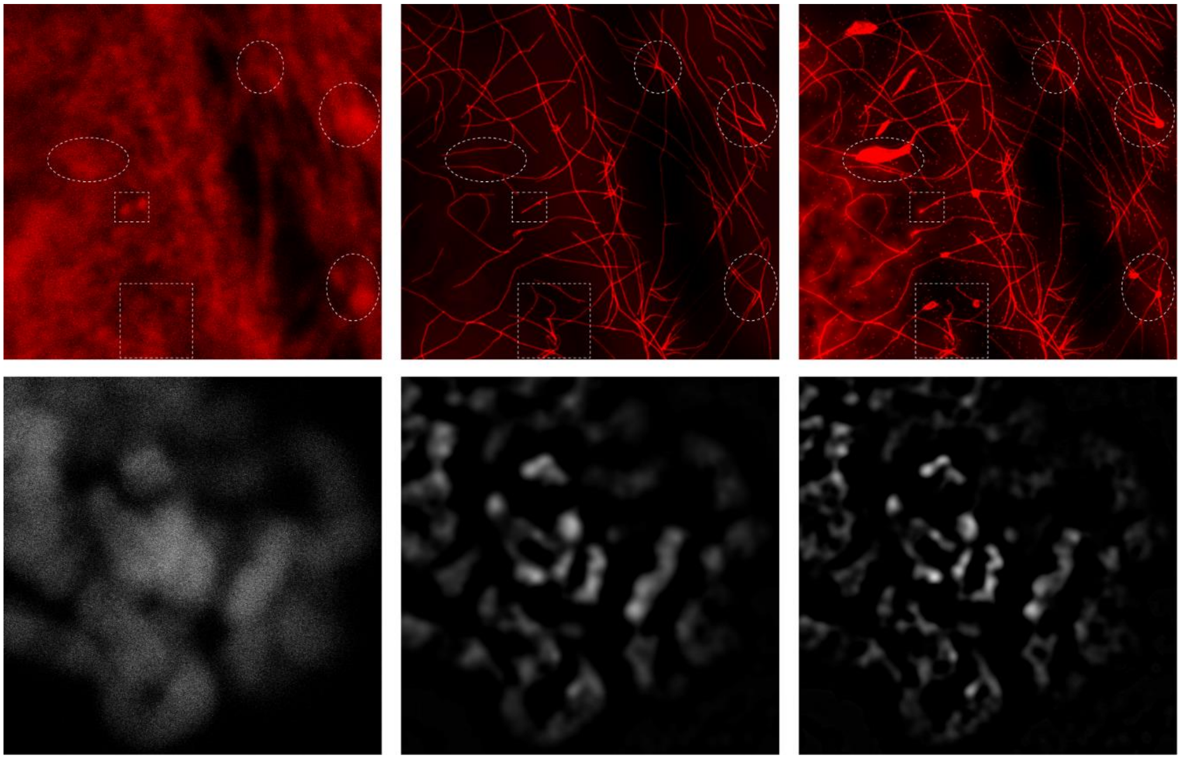

Supplementary Figure S5

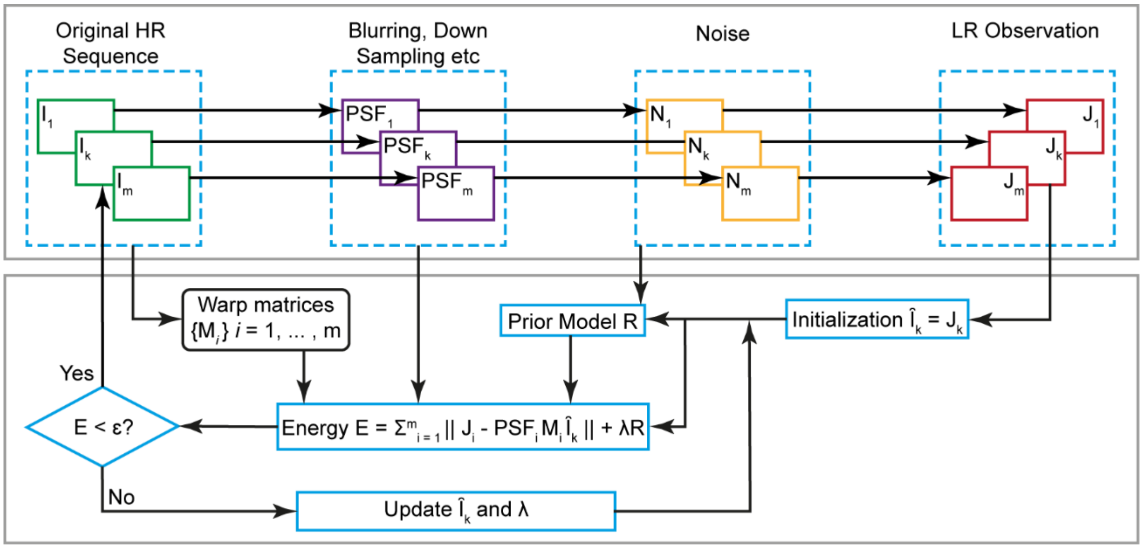

Supplementary Figure Captions

Supplementary Figure S1. Test results on a 2-D 8-bit resolution chart (1024 x 1024 pixels). (**a-b**) A LR ISO 12233 resolution chart and the restored result by our method. (**c**) The peak signal to noise ratio (PSNR) versus the frame number of LR images used for restoration, noise Std σ_n = 20 and PSF Stds σ_{psf} = 5, 10, 15 pixels. (**d**) A comparison of PSNRs by TRAM, ALG, RSR and ZMT versus the Noise Std when the PSF Stds σ_{psf} are 5, 10, 15, respectively. 64 LR images are used. (**e**) A zoom region of the ground truth. (**f**) A single LR image from the same zoom region, with Gaussian-shape PSF Std σ_{psf} = 10 and AWGN of Std σ_n = 20. (**g**)-(**j**) Restoration results by TRAM, ALG, RSR and ZMT, respectively.

Supplementary Figure S2. Test results on 2-D synthetic cell data. (**a**)-(**b**) A synthetic HR cell image (2312 × 384 pixels) and its LR observation corrupted with noise contamiation of Std σ_n= 20 and PSF blurring of Std σ_{psf} = 31 (pixels). 1-D intensity profiles of the five structures in the images are also plotted as green curves. (**c**) Restored image by TRAM and the intensity profile of the five structures. (**d**) FWHM ratio between the LR and restored images for the five structures, respectively. (**e**) FWHM ratio between the LR and restored structures versus the Std of noise in the observations. The number of LR frames and PSF Std are fixed to be 64 and σ_{psf} = 31 (pixels). (**f**) FWHM ratio between the LR and restored structures versus the number of

LR images for different noise levels in the observations of Std σ_n = 10, 20 and 30, respecitively. The Std of PSF is set to be σ_{psf} = 31 (pixels).

Supplementary Figure S3. Resolution of gated stimulated emission depletion microscopy. a) Top row: Confocal and deconvolved gSTED images of 20nm beads, scale bar 1µm. Bottom row: Zoom area of boxed regions from the same field of view, scale bar 100nm. b) Line intensity profiles along the indicated lines in (a). c) box-and-whisker plots indicating the improved resolution by gSTED (mean FWHM of 44 nm) over confocal (mean FWHM of 204 nm). This gSTED data was obtained with 100% depletion power and 2-6.5ns gating. d) Intensity line profiles of a bead imaged with progressively restricted time gating (100% depletion power used) or e) progressively increasing depletion power (gating set to 2-6.5ns for gSTED imaging).

Supplementary Figure S4. Dependence of the TRAM restoration on its operating conditions. Upper row: A zoom area of the actin filaments from the CLSM image Fig 4a (left) and two TRAM restored images of the same area with high (middle) and low (right) denoising threshold, whilst other parameters remain the same. The latter shows more complex structures, including filament bundles, whilst in the presence of more visible noise. There is a good correlation between these images as marked by boxes. Lower row: A zoom area of the DAPI stained nuclei from the CLSM image Fig 4a (left), two TRAM restored images of the same area with high (middle) and low (right) denoising threshold. The latter reviews more detailed structure of the nuclei.

Supplementary Figure S5. Flow chart of TRAM. Upper box depicts the imaging model (eqn. 1) and the lower box describes the iterative energy minimization process (eqn. 2).
